# Safety Assessment of Ubiquinol Acetate: Subchronic Toxicity and Genotoxicity Studies

**DOI:** 10.1155/2019/3680757

**Published:** 2019-04-01

**Authors:** Gajanan Deshmukh, Suresh B. Venkataramaiah, Chandrashekar M. Doreswamy, Mohan C. Umesh, Rajesh B. Subbanna, Bikram K. Pradhan, Srinivas Seekallu, Rajan Sekar, Karthick Prabhu, Sathish Sadagopan, Shri Natrajan Arumugam, Satish Sharma, Govindarajulu Gavara, Selvakumar Balaraman, Ganesh Sambasivam, Ravindra K. Chandrappa, Sarah Flynn, Prasad Shivarudraiah

**Affiliations:** ^1^Anthem Biosciences Pvt. Ltd., #49, F1 & F2, Canara Bank Road, Bommasandra Industrial Area, Phase 1, Bommasandra, Bengaluru 560099, Karnataka, India; ^2^Glanbia plc, Glanbia House, Ring Road, Kilkenny, Ireland

## Abstract

Coenzyme Q10 (CoQ10) is a lipid soluble, endogenous antioxidant present at highest levels in the heart followed by the kidney and liver. The reduced CoQ10 ubiquinol is well known for its chemical instability and low bioavailability. The present study was designed to synthesize ubiquinol acetate, which is more stable and biologically active, and further evaluate its safety and genotoxic potential. Synthesized ubiquinol acetate showed better stability than that of ubiquinol at the end of 3 months. In vitro genotoxicity studies (AMES test, in vitro micronucleus and chromosomal aberration) showed ubiquinol acetate as nongenotoxic with no clastogenic or aneugenic effects at high dose of 5000 and 62.5 *μ*g/mL, respectively. In subchronic toxicity study, ubiquinol acetate was administered orally to Sprague Dawley rats at 150, 300, and 600 mg/kg/day for 90 days. No treatment related adverse effects were observed in males at 600 mg/kg/day; however, females showed treatment related increase in AST and ALT with small focal irregular white-yellow spots in liver on gross necropsy examination. Histopathological evaluation revealed hepatocellular necrosis in high dose females which was considered as adverse. Based on the results, the No-Observed-Adverse-Effect Level (NOAEL) of ubiquinol acetate in males and females was determined as 600 and 300 mg/kg/day, respectively.

## 1. Introduction

Coenzyme Q10 (CoQ10) is a naturally occurring endogenous lipophilic antioxidant present in tissues and food. Meat and fish are the richest animal sources of CoQ10. Oils are the richest vegetable sources with concentrations ranges from 100 to 280 mg/kg in soybean, corn, and olive oil. Nuts and cereals are also known to contain low quantity of CoQ10 [1, 2]. The total dietary intake of CoQ10 ranges from 3 to 6 mg/day in developed countries. Coenzyme Q10 is a cofactor for the ATP synthesis and is part of electron transfer chain in the mitochondria [3]. It exists predominantly in two forms, reduced form as ubiquinol and oxidized form as ubiquinone. Ubiquinol is predominant in the mammalian body and has more bioavailability compared to ubiquinone [4]. About 80 percent of Coenzyme Q10 in healthy individuals exists in the reduced form. Ubiquinol is the strongest lipid soluble antioxidant that is biosynthesized or produced naturally by the human body. Low levels of CoQ10 are associated with aging and several age related pathologies. Exogenous oral supplementation in humans has indirect evidence that the CoQ10 may be incorporated into mitochondria in conditions of CoQ10 tissue deficiency which may enhance electron transfer and ATP synthesis with improvement of age related pathological conditions [5]. Oral CoQ10 has been shown to ameliorate cardiac contractibility and endothelial dysfunction in patients with stable moderate congestive heart failure [6]. Lipid peroxidation is strongly implicated as playing an important role in the development of various pathologies, such as some cardiovascular and neurological diseases [7, 8]. Oxidation of plasma lipoproteins appears to represent a crucial step in thermogenesis and is also likely to occur in other diseases linked to increased free radical production [9]. Being an antioxidant, the CoQ10 is known to prevent initiation and prolongation of lipid peroxidation and protection from further adverse effects by quenching of produced free radicals [10].

In recent years, the use of CoQ10 as nutritional supplement has increased tremendously due to high popularity and being freely available as dietary supplement. The recommended daily intake of CoQ10 in humans has not been determined by any legislation and limited relevant research has been done regarding the dose and bioavailability [11].

Ubiquinol is known for its chemical instability and is rapidly oxidized to ubiquinone in atmospheric oxygen [12]. Hence, compositions containing unprotected ubiquinol require provision of protective packaging or special handling necessary to preserve their action, which is too costly to be commercially feasible on a large scale. Because of this inherent instability, it is not likely to deliver their complete biological potential and the true scope of its utility has not yet fully been realized. Hence to improve the stability and enhance biological activity which is commercially viable, we have designed and synthesized ubiquinol acetate to overcome the drawbacks of ubiquinol.

Most of the reported animal safety studies have been done using ubiquinone or ubiquinol. Since the intended use of the nutritional supplement in humans is for a longer duration, the synthesized ubiquinol acetate was evaluated for its toxic potential by* in vitro* genotoxicity studies and* in vivo *repeated dose subchronic toxicity study by oral (gavage) route in Sprague Dawley rats.

## 2. Materials and Methods

### 2.1. Synthesis and Stability

To overcome the drawbacks of ubiquinol, ubiquinol acetate was synthesized by reduction of ubiquinone using sodium borohydride in ethanol and acetylation with acetic anhydride in the presence of pyridine and a solvent such as n-heptane. The compounds (ubiquinol & ubiquinol acetate) packed in amber colored bottles were tested for its stability at room temperature by HPLC method.

### 2.2. Genotoxicity Studies

The genotoxicity studies, i.e., bacterial reverse mutation test, in vitro chromosomal aberration test, and* In vitro* mammalian micronucleus test, were conducted on ubiquinol acetate as per the respective OECD test guideline and method described by Kothari [13].

#### 2.2.1. Bacterial Reverse Mutation Test

The mutagenicity potential of ubiquinol acetate was evaluated by preincubation method and plate incorporation method using* Salmonella typhimurium *strains TA 1535, TA 1537, TA 98, TA 100, and TA 102. The study was performed in accordance with Bacterial Reverse Mutation Test, OECD TG 471. The mutagenicity assay was executed in two independent assays in the absence and presence of rat liver S9. The assay was carried out in triplicate for every tester strain. The test items that were insoluble in aqueous solutions were diluted to different concentrations using hexane. Ubiquinol acetate was tested at dose levels of 50.1, 158.4, 500.7, 1582.2, and 5000 *μ*g/plate for both preincubation and plate incorporation method. Sodium azide (3 *μ*g/plate) was used as positive control for TA100 and TA1535, 2-nitrofluorene (5*μ*g/plate) for TA98, ICR-191 (2 *μ*g/plate) for TA1537, and cumin hydroperoxide (50 *μ*g/plate) for TA102 strain for assays where S9 activation was not required. In additions, 2-aminoanthracene (10 *μ*g/plate) for TA102 and 2-aminoanthracene (5 *μ*g/plate) for TA98, TA100, TA1535, and TA1537 served as positive mutagens for strains requiring S9 metabolic activation. Hexane was used as the negative control.

A dose range finding study was performed with* Salmonella typhimurium *TA100 strain to determine the noncytotoxic concentration for the main mutagenicity study. Cytotoxicity was defined as a clearing or diminution of the background lawn, the appearance of microcolonies, and/or a decrease of >50% in the number of colonies compared with the hexane vehicle control. Ubiquinol acetate was not cytotoxic at highest concentration of 5000 *μ*g/plate with tested strain in both preincubation and plate incorporation methods with and without S9 metabolic activation condition. Based on the cytotoxicity test results, 5000*μ*g/plate was selected as highest concentration for mutagenicity study with all five strains including TA98, TA100, TA102, TA1535, and TA1537* Salmonella typhimurium* strains. Ubiquinol acetate was tested at dose levels of 50.1, 158.4, 500.7, 1582.2, and 5000 *μ*g/plate for both preincubation and plate incorporation methods.

For preincubation method, different concentrations of ubiquinol acetate (158.4, 500.7, 1582.2, and 5000 *μ*g/plate) or positive control, or vehicle control in the presence or absence of S9 fraction, were mixed with the tester strain and incubated for 30 minutes, mixed with top agar containing Histidine + Biotin, and poured onto agar plates containing minimal glucose agar. After solidification, the plates were incubated for 48–72 h. For plate incorporation method, different concentrations of test item, positive control or vehicle control, tester strain S9 mix/phosphate buffer were added onto top agar tubes containing Histidine + Biotin. The mixture was mixed gently and plated onto agar plates containing minimal glucose medium. After solidification, the plates were incubated at 37°C for 48-72 hours.

#### 2.2.2. In Vitro Chromosomal Aberration Test

The genotoxic potential of ubiquinol acetate to induce structural and numerical chromosome aberrations was evaluated in Chinese Hamster Ovary cells (CHO-K1) as test system. The study was performed in accordance with OECD Guideline for Testing of Chemicals # 473 (In Vitro Mammalian Chromosomal Aberration Test, adopted on 29^th^ July 2016) in a GLP compliant laboratory. As the test system, CHO-K1 cells were procured from ATCC. Liquid nitrogen cryopreserved cell stocks of CHO-K1 cells (passage no 6) were revived and cultured in Ham's F-12K culture medium supplemented with 10% fetal bovine serum, 1.0 mM L-glutamine, 100 IU/mL penicillin, and 100 *μ*g/mL streptomycin. The cell cultures were prepared in T75 cm^2^ culture flasks and were incubated at 37°C and 5% CO_2_.

A dose range finding study of test item was performed to measure the cytotoxicity in CHO-K1 cells and also to select the dose levels for the main study to evaluate its clastogenic potential. CHO-K1 cells were grown in 6 well culture plates (20 hours after seeding) and were exposed to 6 concentrations (n=1) of test item ranging from 62.5 to 1.953 *μ*g/mL in presence (4 hours) and absence (4 and 24 hours) of metabolic activation system. The test medium with metabolic activation system consisted of 1% (v/v) rat liver S9 prepared from male Sprague Dawley rats treated with Aroclor-1254 (Molecular Toxicology, Inc., USA), 10 mM potassium phosphate, 0.5 mM glucose 6 phosphate, 0.4 mM nicotinamide adenine dinucleotide phosphate, 3.3 mM potassium chloride, and 0.8 mM magnesium chloride. The concentrations of test item were chosen based on its solubility in Ham's F-12K culture medium. The test item stocks were prepared in acetone and used at a final acetone concentration of 1% (v/v) in the test medium. The acetone stocks and the test media were prepared freshly and used immediately for treating the cells. Vehicle control cultures treated with 1% acetone were included at each incubation condition. The cultures treated for duration of 4 hours were washed with 2 mL of sterile 1X phosphate buffer saline and added with 2 mL of 10% fetal bovine serum containing culture medium. Cultures were harvested by trypsinization after 24 hours of initiation of the treatment. The cultures receiving the continuous treatment (24 hours) were left undisturbed till the harvest. After harvesting, the viable cells were counted by trypan blue exclusion method for each treated culture and % Relative Increase in Cell Count (RICC) and % cytotoxicity were determined using the formula:

(1) $$\begin{array}{c} RICC\left(\;\%\;\right)=\frac{Increase\;\;in\;\;No.\;\;of\;\;cells\;\;in\;\;treated\;\;cultures\;\;\left(Final-Starting\right)X\;\;100}{Increase\;\;in\;\;No.\;\;of\;\;cells\;\;in\;\;control\;\;cultures\;\;\left(Final-Starting\right)} \end{array}$$

Main study was performed using 3 concentrations of test item (n=2) for which the concentrations were selected based on the dose range finding study. The selected dose levels were 62.5 *μ*g/mL (high dose), 31.25 *μ*g/mL (mid dose), and 15.63 *μ*g/mL (low dose) and were separated by factor of 2. The preparation of doses, incubation conditions, and durations were similar to the dose range finding study except that all the cultures were added with metaphase arresting agent Colcemid® (0.2 *μ*g/mL) 2-3 hours before harvest. Positive controls known to induce structural aberrations in CHO-K1 cells such as Mitomycin C (0.4 *μ*g/mL) and Cyclophosphamide (5 *μ*g/mL) were included alongside vehicle and media controls. Upon harvest, the small aliquots of cells from each treatment culture were counted to determine the cytotoxicity. Harvested cells were treated with hypotonic solution of 0.8% (w/v) trisodium citrate for 12-15 minutes at 37°C and later the cells were fixed with methanol and acetic acid fixative (3:1). The cells stored at 5 ± 3°C were pelleted down by centrifuging at 200* g* for 10 minutes at 4°C. Pelleted cells were resuspended in small volume of fixative and 20-30 *μ*L cell suspension was dropped on to grease free, clean, and methanol chilled glass slides held in an inclined angle from a height of 10-15 cm using a micropipette. Slides were dried and stained with 5% Giemsa stain. Stained slides were permanently mounted using DPX mountant media and coverslips, allowed to dry, and coded to mask the identity.

Slides were examined under the compound microscope at 1000X magnification. Well spread metaphases with 20 ± 2 chromosomes were considered for scoring structural aberrations. Minimum 300 metaphases equally divided among the replicates were scored for chromosomal aberrations. When clear positive responses with increased frequency of aberrations were observed, the scoring number was reduced. The percentage of cells with structural chromosomal aberration(s) was evaluated. Chromatid and chromosome-type aberrations classified by subtypes (breaks, exchanges) were listed separately with their numbers and frequencies for experimental and control cultures. Gaps were recorded and reported separately but not included in the total aberration frequency. The observations other than structural aberrations such as polyploidy and endoreduplication were tabulated, but they were not included in calculating the % of cells with chromosomal aberrations. Statistical analysis was performed on the obtained results by Fisher's exact test for pair wise comparisons between each treated and vehicle control groups using GraphPad Prism 5.03 statistical software package. The significance level was chosen at P< 0.05.

#### 2.2.3. In Vitro Mammalian Micronucleus Test

The potential of ubiquinol acetate to induce clastogenicity/aneugenicity by measuring the extent of micronucleus formation in the cytoplasm of interphase cells was evaluated in Chinese Hamster Ovary cells (CHO-K1) as the test system. The study was performed in accordance with the OECD Guideline for Testing of Chemicals # 487 (In Vitro Mammalian Cell Micronucleus Test, adopted on 29^th^ July 2016) in a GLP compliant laboratory. Chinese Hamster Ovary cells (CHO-K1) with epithelial like cell morphology and modal chromosome number of 20±2 were used. CHO-K1 cells were procured from ATCC, prorogated, and preserved as cryostocks. Cryopreserved stocks of CHO-K1 cells (passage no 6.) were revived and cultured in Ham's F-12 supplemented with 10% fetal bovine serum, 1.0 mM L-glutamine, 100 IU/ml penicillin, and 100 *μ*g/mL streptomycin. The cultures were grown in a 5% CO_2_ atmosphere at 37°C.

The dose range finding study was performed to determine the cytotoxicity of test item in CHO-K1 cells and also to select the appropriate dose levels to evaluate the clastogenic potential during the main study. In dose range finding study, CHO-K1 cells were treated with eight different test concentrations ranging from 62.5 to 0.488 *μ*g/mL both in presence (4 hours) and absence (4 and 24 hours) of metabolic activation system. The test medium with metabolic activation system consisted of 1% (v/v) rat liver S9 prepared from male Sprague Dawley rats treated with Aroclor-1254 (Molecular Toxicology, Inc., USA), 10 mM potassium phosphate, 0.5 mM glucose 6 phosphate, 0.4 mM nicotinamide adenine dinucleotide phosphate, 3.3 mM potassium chloride, and 0.8 mM magnesium chloride. The 100 times higher acetonitrile stocks of the test item were spiked into culture media at 1% to get the desired concentrations. Vehicle controls containing 1% acetone and media controls were included in all the treatment conditions. All the cultures were treated with cytokinesis blocker Cytochalasin B (4 *μ*g/mL) 20 hours before harvest. The cells were harvested after 24 hours of initiation of the test item treatment. Harvested cells were treated with hypotonic solution 1% (w/v) trisodium citrate for 10 minutes and centrifuged at 200 g for 10 minutes to pellet the cells. Pelleted cells were fixed using methanol and acetic acid fixative (3:1) and were stored at 4°C for overnight. The cells were again pelleted down and resuspended in small volume of cold fixative and dropped onto clean chilled slides. Slides were stained using Wright Geimsa for 20 minutes. Stained dry slides were permanently mounted using DPX (Sigma Aldrich) mountant and coverslips. Slides from each culture were scored under compound microscope for mono-, bi-, and multinucleated cells. The cytotoxic or cytostatic activities of the treated culture were estimated by comparing the cytokinesis-block proliferation index (CBPI) values with CBPI values of control cultures as per the following equations:

(2) CBPI=No.  mononucleated  cells+2  ×  No.  binucleated  cells+3  ×  No.  multinucleated  cellsTotal  number  of  cells%  Cytostasis=100-100CBPIT−1÷CBPIC−1

  where T = test item treatment culture
  C = control culture

In main study 3 dose levels of low (15.625 *μ*g/ml), mid (31.25 *μ*g/ml), and high (62.5 *μ*g/ml) were tested in duplicate cultures in presence (4 hours) and absence (4 and 24 hours) of metabolic activation system. In addition to test item and negative control groups (media and vehicle), positive control group such as Mitomycin C at 0.5 *μ*g/ml in absence of metabolic activation and Cyclophosphamide at 5 *μ*g/ml in presence of metabolic activation system were included. All the treatment procedures, harvesting and slide preparations, were carried out as described in the dose range finding except in the main study along with CBPI to estimate the cytostasis; the slides were scored for presence of micronucleus to determine the clastogenic potential. Statistical analysis was performed on the obtained results by Fisher's exact test for pair wise comparisons between each treated and vehicle control groups using GraphPad Prism 5.03 statistical software package. The significance level was chosen at P< 0.05.

### 2.3. Subchronic Study

#### 2.3.1. Study Compliance

The subchronic toxicity study of ubiquinol acetate was performed in accordance with OECD Principles on Good Laboratory Practice [C (97)186/final], OECD Guideline for Testing of Chemicals: Repeated Dose 90-Day Oral Toxicity Study in Rodents 408 (adopted 21^st^ September 1998), standard operating procedures at Anthem Biosciences Pvt. Ltd., Bangalore, India, methods as described [13] and as per the mutually agreed study plan with the sponsor. The study protocol was approved by Institutional Animal Ethics Committee (IAEC Protocol No.: ABD/IAEC/PR/33R6/15-16) on 09^th^ Jan, 2017, as per the recommendations of the Committee for the Purpose of Control and Supervision of Experiments on Animals (CPCSEA) guidelines for laboratory animal's facility published in the gazette of India, December 15, 1998. The study was conducted in animal facility at Anthem Biosciences which is AAALAC (Association for Assessment and Accreditation of Laboratory Animal Care) and GLP accredited.

#### 2.3.2. Test Item

The test item, ubiquinol acetate used in the present studies, was synthesized and characterized by Department of Chemistry, Anthem Biosciences Pvt. Ltd., Bengaluru, India [Table 1, specifications & certificate of analysis of ubiquinol acetate].

The test item ubiquinol acetate (Lot no. A31600427) is white to off-white color powder with assay purity (HPLC % w/w) of 99.1%. The test item was stored in amber colored bottle at room temperature (19-25°C) with a shelf life of 3 years.

#### 2.3.3. Animals

Sprague Dawley rats procured from registered CPCSEA vendor (Vivo Bio Tech Ltd., Telangana, India) were used for this study. Veterinary examination of all the animals was recorded on the day of receipt and they were quarantined for a period of six days. Once the animals in the quarantine room were declared healthy by the veterinarian, the animals were shifted to the experimental room for acclimatization. One hundred rats were divided into 4 main study groups with 10 animals/sex and 2 satellite/recovery groups with 5 animals/sex. The average age of animals at the time of treatment was 5-6 weeks old. Maximum of three animals were housed in a standard polycarbonate cage (size: L 42.1 x W 29 x H 19 cm) with stainless steel mesh top grill with facilities for holding pelleted food and drinking water in water bottle fitted with stainless steel sipper tube. Clean autoclaved corncob was provided as bedding material. All the animals were housed under standard laboratory conditions, in environmentally monitored air-conditioned room with adequate fresh air supply (10-15 air changes per hour), room temperature of 22°C ± 3°C, and relative humidity of 30-70 %, with 12 hours light and 12 hours dark cycle. Temperature and humidity were recorded once daily throughout the experimental period.

The animals were fed* ad libitum* throughout the acclimatization and experimental period except for a period of fasting. Autoclaved standard rodent feed (Amrut, manufactured by Pranav Agro Industries Ltd.) was provided with ad libitum water throughout the acclimatization and experimental period. Water from aqua guard water filter cum purifier passed through ultraviolet was autoclaved and provided in autoclaved polypropylene water bottles.

#### 2.3.4. Treatment

Animal grouping was done according to body weight stratification and randomization. Body weight of the animals after grouping was recorded and analyzed statistically to rule out the statistical significant difference between groups within each sex. Corn oil was used as a vehicle for test item formulation. Main study group animals were divided into four groups (10 animals/sex/group) and were administered orally (gavage) once daily with corn oil alone for vehicle control group (G1- vehicle control) and test item formulation at dose levels of 150 (G2- low dose), 300 (G3- mid dose), and 600 mg/kg body weight (G4- high dose) at a dose volume of 4 mL/kg body weight for 90 consecutive days. Satellite group animals were divided into two groups (5 animals/sex/group) and were administered orally (gavage) once daily with corn oil alone for vehicle control recovery group (G1R-vehicle control) and test item formulation at dose level of 600 mg/kg body weight (G4R - high dose recovery) at a dose volume of 4 mL/kg body weight for 90 consecutive days. Satellite group animals were kept for observation for 14 days after withdrawal of the treatment. Stability of the test item in the vehicle was found to be stable as per method validation study up to 6 hours at room temperature (22±3°C) and seven days when stored at 2-8°C. Based on the stability results, the prepared formulation was stored at 2-8°C and used within 7 days for administration. The actual dose volume for each animal was calculated based on the recent nonfasted weekly body weight of the animals.

#### 2.3.5. Observations & Parameters Evaluated

*(1) Homogeneity and Dose Confirmation Analysis of the Test Formulation.* The test item formulations at concentrations of 37.5, 75.0, and 150 mg/mL were assessed for homogeneity (3 layers, top, middle, and bottom) and dose confirmation analysis by validated HPLC method on days 0, 49, and 90 of the treatment period as per method validation study. Corn oil samples which were used as vehicle were analyzed for active ingredients to rule out any possible contamination of the test item. Chromatography was carried out on a Luna C18(2)100Å, 150X4.6mm, 5*μ*m column with a mobile phase consisting of Methanol: 2 Propanol (50:50 v/v). Detection was carried out by PDA at a wavelength 206 nm. The compound was eluted isocratically at a steady flow rate of 1.0 mL/min and the analyte ubiquinol acetate retention time was at 6.0 min with an asymmetry factor of 1.70.

*(2) Mortality, Clinical Signs Examination, Body Weight, and Feed Consumption.. *All animals were observed once daily for clinical signs of toxicity and twice daily for mortality and morbidity. The animals were subjected to detailed clinical examinations before initiation of the treatment and weekly thereafter (± 1 day) during the study. Individual animal body weights were recorded on receipt to experimental room, on the day of initiation of treatment and weekly thereafter (± 1 day) during the study period. Fasting body weights were recorded at terminal sacrifice. Individual animal food consumption was recorded weekly (± 1 day) and group mean food consumption (g/animal/day/week) was calculated.

*(3) Neurological/Functional & Ophthalmological Examination.. *Neurological/functional examination was carried out during the 13^th^ week for vehicle control (G1) and high dose (G4) group and the 15^th^ week for vehicle control recovery (G1R) and high dose recovery (G4R) group animals. The neurological examinations such as home cage (posture, respiratory pattern, vocalization, palpebral closure), handheld (ease of removal, ease of handling, lacrimation, salivation, chromodacryorrhea, hair coat, muscle tone), open field (clonic/tonic involuntary movement, gait, mobility, arousal, stereotype, piloerection, exophthalmos, number of defecations, number of urinations, number of rearing), reflex measurements (approach response, touch response, auditory response, pupil reflex, corneal reflex, air righting reflex, pinna reflex), neuromuscular (forelimb grip strength test, hind limb foot splay and physiological measurements (rectal temperature)) were performed. Ophthalmological examination was performed on all animals before the treatment, the 13^th^ week of the study in vehicle control (G1) and high dose (G4) group animals and the 15^th^ week of the study in vehicle control recovery (G1R) and high dose recovery (G4R) group animals. Mydriasis was induced using topical agent (1% Tropicamide) and examined by indirect ophthalmoscope. If treatment related changes were observed in neurological and ophthalmological examination in high dose group animals (G4), the examination will be extended to low (G2) and mid dose (G3) group animals.

#### 2.3.6. Clinical Pathology

The animals were fasted overnight before urine and blood collection. Water was provided ad libitum during fasting period. Animals were housed in metabolic cages with collection containers containing thymol as preservative for urine collection. Blood samples were collected from the all surviving rats of each sex per group from retro-orbital plexus using fine glass capillary under mild isoflurane anesthesia. Blood samples for the hematology and clinical chemistry were collected in prelabeled tubes containing K_2_EDTA (2 mg/mL) and lithium heparin (20 IU/mL), respectively.

*(1) Urinalysis. . *Urine samples were collected during the last week of the study (week 13) and satellite groups on week 15. An aliquot of urine samples was centrifuged at 1500 rpm for 10 min at 4°C ± 2°C for urine microscopy examination. Parameters like color, appearance, volume, and microscopic examination of urine sediment (Epithelial cells, RBC, Casts, Crystals, Pus cells, and Bacteria) were recorded. Biochemical parameters like specific gravity, pH, occult blood, bilirubin, urobilinogen, ketone bodies, protein, and glucose were analyzed using Clinitek Status+ urine analyzer.

*(2) Hematology. . *Blood samples were collected on day 91 for main study group and on day 105 for the satellite group animals. The following hematology parameters were analyzed using ADVIA 2120 (Siemens Limited) Hematology analyzer: hematology parameters, hemoglobin (HB), hematocrit (HCT), total erythrocyte count (RBC), total leukocyte count (WBC), mean corpuscular volume (MCV), mean corpuscular hemoglobin (MCH), mean corpuscular hemoglobin concentration (MCHC), platelet count, and differential leucocytes count (DLC). Blood clotting time was performed by capillary tube method.

*(3) Clinical Chemistry. . *Blood samples collected were centrifuged at 4000 rpm for 10 minutes at 4±2°C. The following clinical chemistry parameters were analyzed using Siemens Dimension Xpand Plus clinical chemistry analyzer: alanine aminotransferase (ALT), aspartate aminotransferase (AST), alkaline phosphatase (ALP), total protein, albumin, total bilirubin, gamma-glutamyl transferase (GGT), glucose, total cholesterol, creatinine, blood urea nitrogen (BUN), triglycerides, phosphorous, calcium, and Globulin (Calculated). Sodium, potassium, and chloride were estimated using electrolyte analyzer (Siemens Rapidchem RC744).

#### 2.3.7. Pathology

*(1) Gross Necropsy.* At the end of treatment period on day 90 and day 105, all surviving animals of main study groups and satellite groups respectively were fasted overnight. Fasting body weights of the animals were recorded and euthanized using excess dose of carbon dioxide. Detailed gross necropsy, which includes careful examination of the external surface of the body, all orifices, and cranial, thoracic, and abdominal cavities and their contents, was performed. Any abnormal lesions/affected organs found during the gross necropsy were recorded.

*(2) Organ Weights. . *After gross necropsy examination, the following organs from all animals were trimmed off any adherent tissues as appropriate and weighed wet as soon as possible to avoid drying (liver, kidneys, adrenals, spleen, heart, uterus and cervix, epididymides, thymus, brain, testes, ovaries and prostate, seminal vesicles with coagulating glands). Paired organs were weighed together and relative organ weights were calculated against fasting body weight.

*(3) Tissue Collection. . *The following organs were collected and trimmed off any adherent tissue, as appropriate, and were preserved in 10% neutral buffered formalin as fixative (liver, kidneys, adrenals, testes, epididymides, thymus, spleen, brain, heart, uterus and cervix, ovaries, prostate, seminal vesicles with coagulating gland, spinal cord, eyes with optic nerve, stomach, esophagus, aorta, small and large intestine, Peyer's patches, thyroid and parathyroid, trachea, lungs, vagina, urinary bladder, lymph nodes (submandibular and mesenteric), salivary glands, pancreas, skeletal muscle, sciatic nerve, sternum, pituitary, and skin with mammary glands). Eyes and testes collected were preserved in Davidsons' and Modified Davidson's fixative respectively for 24-48 hours and transferred to 10% neutral buffered formalin.

*(4) Histopathological Examination. . *Collected and processed tissues were embedded in paraffin wax, sectioned at five micrometers, and stained with hematoxylin and eosin. The histopathological examination was conducted on the specified list of tissues including all macroscopically abnormal tissues of all animals from vehicle control (G1) and high dose group (G4). On observation of treatment related changes, the examination of the target organs was extended to satellite groups (G1R & G4R), low (G2) and mid dose (G3) of main study groups.

#### 2.3.8. Statistical Analysis

After verification of the raw data, body weight, body weight gain, food consumption, neurological examination, absolute and relative organ weights, urinalysis (applicable parameters), hematology, and clinical chemistry were subjected to statistical analyses using Graph Pad Prism version 5.03, Graph Pad Software. One-way ANOVA with Dunnett's posttest were done for different treatment groups comparing with the respective control group data. Male and female data were considered separately for analysis. Student's t-test was considered for analysis wherever required. All analysis and comparisons were evaluated at the 95% level of confidence (p<0.05).

## 3. Results

### 3.1. Synthesis and Stability

**Figure 1:**
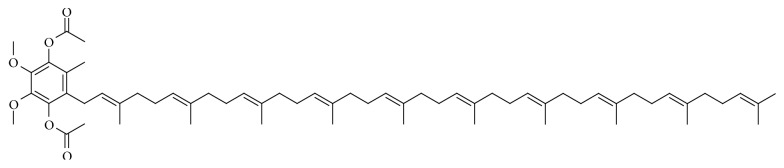
Structure of ubiquinol acetate.

Synthesized ubiquinol acetate was with a HPLC assay purity (w/w) of 99.1%. The structure of ubiquinol acetate is shown in Figure 1 and certificate of analysis (see Table 2).

Stability testing performed at initiation, 1, and 3 months after synthesis at room temperature revealed that the ubiquinol acetate was stable at all time points. Ubiquinol was unstable and showed HPLC purity of 98%, 53%, and 22% stable at initiation, 1, and 3 months after storage at room temperature (see Table 1) due to oxidation.

### 3.2. Genotoxicity Studies 

#### 3.2.1. Bacterial Reverse Mutation Test

In both the preincubation and plate incorporation methods with presence and absence of S9 activation system, no precipitation or cytotoxicity was observed at the tested concentrations with* S. typhimurium* TA100 strain.

The test item is considered mutagenic, if there was a dose related increase over the range tested and/or a reproducible increase at one or more doses in the number of revertant colonies in at least one strain or if the number of revertant colonies in test dose levels is more than two to three folds when compared to vehicle control.

**Figure 2:**
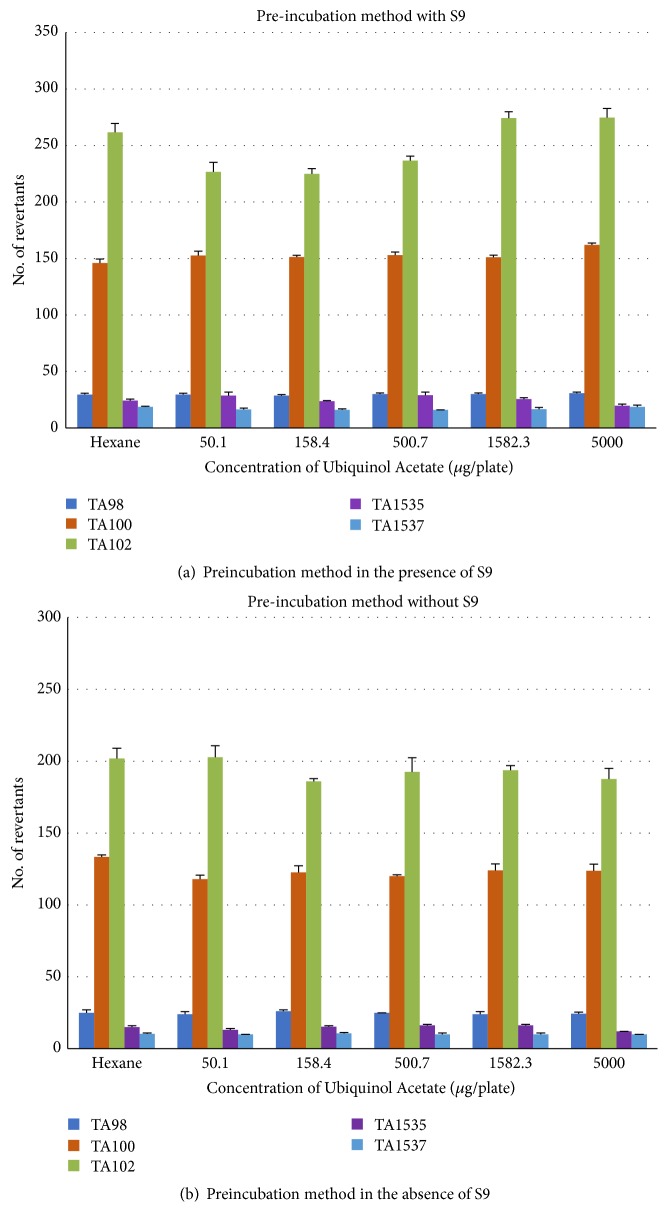
Effect of ubiquinol acetate on number of revertants in preincubation method using* Salmonella typhimurium *tester strains in the presence (a) and absence (b) of metabolic activation. Each concentration was tested in triplicate (n = 3) and the values are represented as mean ± SD.

**Figure 3:**
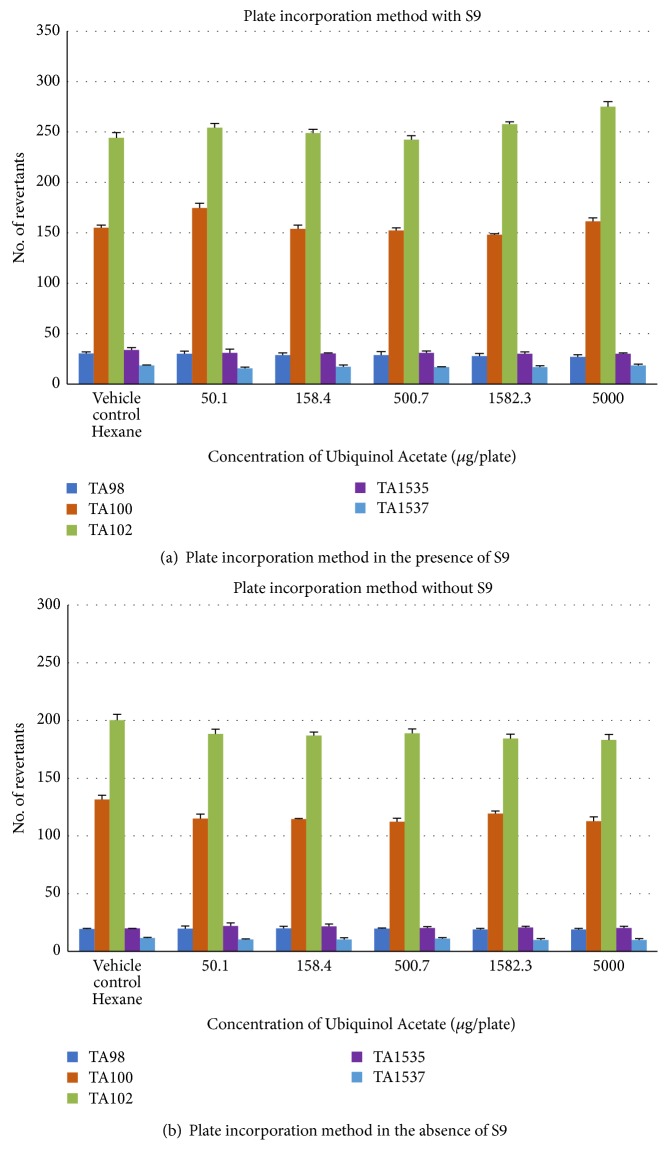
Effect of ubiquinol acetate on number of revertants in plate incorporation method using* Salmonella typhimurium *tester strains in the presence (a) and absence (b) of metabolic activation. Each concentration was tested in triplicate (n = 3) and the values are represented as mean ± SD.

Ubiquinol acetate was not found to be mutagenic in preincubation assay both in the presence (Figure 2(a)) and absence of (Figure 2(b)) S9 metabolic activation with* S. typhimurium* strains TA98, TA100, TA102, TA1535, and TA1537at all tested concentrations (50.1, 158.4, 500.7, 1582.3, and 5000 *μ*g/plate). Ubiquinol acetate was nonmutagenic at all concentrations tested both in the presence (Figure 3(a)) and absence of (Figure 3(b)) S9 metabolic activation when tested by plate incorporation method with all tester strains. The validity of the study was confirmed by more than twofold increase in the number of revertant colonies in positive control plates compared to vehicle control.

#### 3.2.2. In Vitro Chromosomal Aberration Test

Ubiquinol acetate treated cultures were evaluated for cytotoxicity during dose range finding study. About 18.27% and 16.00% of cytotoxicity were observed in cultures treated with 62.5 *μ*g/mL of ubiquinol acetate for 4 hours in presence and absence of metabolic activation system respectively. No considerable amount of cytotoxicity was observed in all other tested concentrations except in 31.25 *μ*g/mL treated cultures where 15% of cytotoxicity was observed in presence of metabolic activation system. Cultures with 24-hour continuous treatment did not show any cytotoxicity.

Based on dose range finding result, 62.5, 31.25, and 15.625 *μ*g/mL of concentrations were selected and evaluated in the main study for induction of chromosomal aberrations in absence and presence of metabolic activation. None of the ubiquinol acetate treated cultures showed considerable amount of cytotoxicity (see Table 4). The percentages of aberrant cells in 4-hour treated cultures in absence of metabolic activation were 0.67, 1 and 0.67% at 15.63, 31.25, and 62.5 *μ*g/mL, respectively. The cells with aberrations observed were statistically nonsignificant (p>0.05) from vehicle control (1% acetone treated cultures) and were within the historical control values. In cultures treated for 4 hours in presence of metabolic activation, observed cytotoxicities were minimal. The percentages of cells with structural aberrations were 1, 2, and 1.67% at 15.63, 31.25, and 62.5 *μ*g/mL of treatment, respectively, and the observed values were nonsignificant (p>0.05) from the vehicle control. No cytotoxicity was observed in 24 hour continuous treated cultures. Cells with structural aberrations observed in 24-hour treatment were 1.33, 0.67, and 1% at 15.63, 31.25, and 62.5 *μ*g/mL, respectively, and were found to be statistically nonsignificant (p>0.05) from vehicle controls. The percentages of structural aberrations observed in the vehicle treated cultures (1% acetone) both in presence and absence of metabolic activation were not significant from the media controls and were within the historical control values. Reference items (positive controls) showed statistically significant increase in the percent aberrant cell there by fulfilling the positivity criteria and the results observed were within the historical range for positive controls.

#### 3.2.3. In Vitro Mammalian Micronucleus Test

In dose range finding study, no cytotoxicity was observed in any of the 8 tested concentrations of ubiquinol acetate (62.5–0.488 *μ*g/mL) in CHO-K1 cells in comparison to vehicle control, both in presence (4 hours treatment) and absence (4 and 24 hours treatment) of metabolic activation system. The cytotoxicity was estimated by measuring the cytokinesis-block proliferation index (CBPI).

During main study 3 concentrations of ubiquinol acetate at 15.625, 31.25, and 62.5 *μ*g/mL were tested as low, mid, and high dose levels for evaluating the clastogenicity by micronucleus test in both presence and absence of metabolic activation system. Cell cultures treated with media and vehicle controls (1% acetone) in absence of metabolic activation for 4 hours showed 0.6 and 0.85% of micronucleus frequency; at similar conditions test item treated cultures showed 0.70%, 0.85%, and 0.85% of micronuclei frequency at low, mid, and high dose levels, respectively. The values observed from the test item treatment were statistically nonsignificant from the vehicle control (p>0.05). Positive control Mitomycin C at 0.5 *μ*g/mL showed 9.4% micronuclei frequency and was statistically significant from the vehicle control (p<0.05). No considerable amount of cytotoxicity was observed in any of the test item treated cultures for 4 hours in absence of metabolic activation. Cultures treated with media and vehicle controls for continuous 24 hours in absence of metabolic activation showed 0.8 and 0.45% of micronuclei frequency; test item treated cultures at similar conditions showed 0.70%, 0.60%, and 0.65% of micronuclei frequency in low, mid, and high dose levels, respectively, and were found to be statistically nonsignificant from the vehicle controls (p>0.05). Positive control Mitomycin C at 0.5 *μ*g/mL showed 10.65 % micronuclei frequency and was statistically significant from the vehicle control (p<0.05). No considerable amount of cytotoxicity was observed in any of the test item treated cultures for 24 hours in absence of metabolic activation. Cultures treated with media and vehicle controls for 4 hours in presence of metabolic activation showed 0.6% and 0.5% of micronuclei frequency; test item treated cultures at similar conditions showed 0.70%, 0.85%, and 0.85% of micronuclei frequency in low, mid, and high dose levels, respectively, and were found to be statistically nonsignificant from the vehicle controls (p>0.05). Positive control Cyclophosphamide at 5 *μ*g/mL showed 7.20% micronuclei frequency and was statistically significant from the vehicle control (p<0.05). No considerable amount of cytotoxicity was observed in any of the test item treated cultures for 4 hours in absence of metabolic activation. The micronuclei frequency observed in the vehicle control (1% acetone) was not significantly different from the media control and the values observed were found to be within the historical control values for negative controls. The results of in vitro micronucleus study are presented in Table 5.

### 3.3. Subchronic Toxicity Study

#### 3.3.1. Homogeneity and Dose Conformation Analysis

Validated HPLC method was employed to quantify the ubiquinol acetate in dose formulations.

A calibration curve at concentrations from 10.020 to 120.234 *μ*g/mL was found to be linear and the correlation coefficient (r^2^) was greater than 0.98.

The active ingredients in the prepared formulations were found to be homogeneously distributed in the vehicle. The vehicle control sample analysis at all the intervals of dose confirmation analysis was not showing any peak interference at the retention time of main peak, which indicates that there was no contamination of the vehicle with the test item. The results of dose confirmation analysis at different intervals analyzed during the treatment period were within the acceptance limits of variation from the nominal concentrations (±15%) (see Table 3).

#### 3.3.2. Survival, Clinical Observations, Body Weights, and Feed Consumption

**Figure 4:**
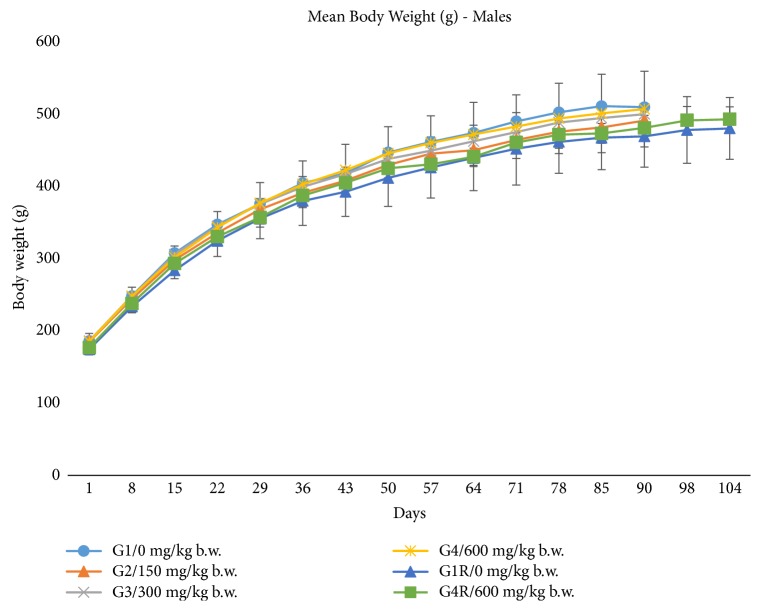
Effect of ubiquinol acetate on body weights in male rats. Mean body weights for male rats during a 90-day oral (gavage) toxicity study. The values are presented as means ± standard deviation (10 rats/sex/group for main study group and 5 rats/sex/group for recovery group).

**Figure 5:**
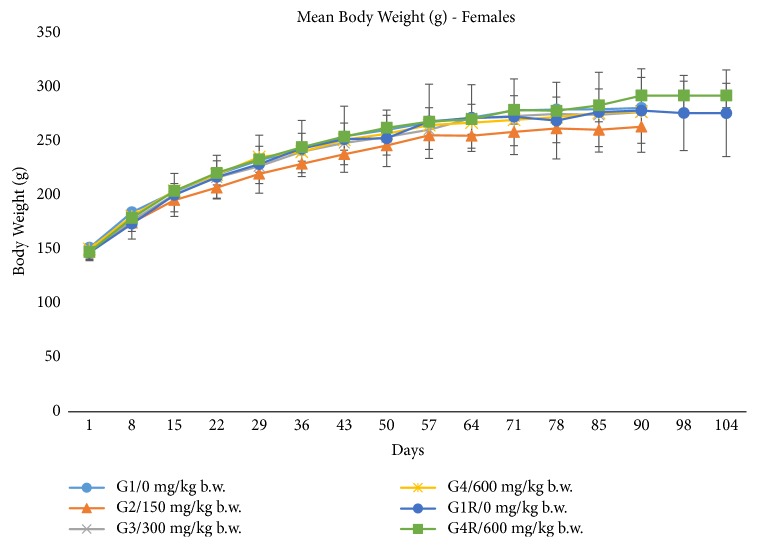
Effect of ubiquinol acetate on body weights in female rats. Mean body weights for female rats during a 90-day oral (gavage) toxicity study. The values are presented as means ± standard deviation (10 rats/sex/group for main study group and 5 rats/sex/group for recovery group).

Animals administered with the vehicle and test item formulation were found to have no adverse clinical signs of toxicity and mortality/morbidity till the scheduled sacrifice. The body weight and percent body weight gain had no adverse effects in both sexes throughout the experimental period and were comparable to the respective vehicle control group. There was no statistically significant treatment related changes in body weights (see Figures 4 and 5) and percent body weight gain in both sexes (see Table 6). The weekly average food consumption (g/rat/day) showed no treatment related adverse effects in both sexes and was found to be comparable to respective control groups (data not shown). The results of weekly feed consumption obtained were statistically nonsignificant and were in accordance with the findings reported by Kitano [14].

#### 3.3.3. Neurological/Functional & Ophthalmological Examination

Neurological/functional examination and ophthalmological examination carried out during the 13^th^ (G1 & G4) and 15^th^ weeks of the study (G1R & G4R) were found to be comparable with the concurrent vehicle control group. No treatment related neurological and ocular abnormalities were observed; hence, the examination was not extended to low dose (G2) and mid dose (G3) groups of the main study.

#### 3.3.4. Clinical Pathology

*(1) Hematology.* There were no treatment related adverse effects observed in hematology parameters of any of treated groups as compared to concurrent vehicle control. However, statistically significant decrease in hemoglobin (G4M), hematocrit (G3F, G4F, G4M), and MCV (G2F, G3F, G4F) and significant increase in MCHC (G2F, G3F, G4F) and blood clotting time (G4RF) were observed when compared to concurrent control group (see Tables 7 and 8).

*(2) Clinical Chemistry. . *There were treatment related significant increase in AST (G4F) and ALT (G3F, G4F, G4RF) at the end of 90 days of treatment in high dose females and also increase in ALT in females of high dose recovery groups. The observed changes were correlated to histological changes in liver, which were considered to be treatment related and can be considered adverse.

However, significant increase in calculated globulin (G4M), bilirubin (G4M), total protein (G3M, G4M), and creatinine (G2M, G4RF) and statistical significant decrease in sodium (G4RM) and albumin (G4RF) were observed which were found to be either non-dose-dependent, observed only in one sex, no correlated adverse histological changes, and the changes were not observed in the recovery groups. Hence, these variations were considered to be incidental and not treatment related (see Tables 9 and 10).

*(3) Urinalysis. . *The urinalysis parameters analyzed of main study (day 91) and recovery groups (day 105) of both sexes were found to have no treatment related adverse effects and were comparable with the concurrent control groups.

#### 3.3.5. Organ Weights

There was no treatment related toxicologically significant changes in absolute and relative organ weights when compared to the concurrent vehicle control group. However, statistically significant increases in absolute and relative weight of thymus (G4M), prostate, seminal vesicles with coagulating gland and kidneys (G4RM) were observed in males compared to concurrent vehicle control group. No significant changes were observed in any of the organs in treated females as compared to concurrent control groups. The changes in organ weights of males are considered to be incidental due to lack of dose-dependency, sex specific and lack of correlative histopathological changes (see Tables 11 and 12).

#### 3.3.6. Pathology

Treatment related gross pathological changes of small white to yellow irregular focal spots were observed in liver (G4F-2/10) and small yellow foci in lungs (G4M-2/10, G4F-3/10, G4RF-1/5) of high dose treated groups when compared with the concurrent vehicle control group (see Table 13).

On histopathological evaluation, treatment related changes were observed in lungs, liver, and mesenteric lymph nodes of females of high dose group (Table 14). On microscopic examination of high dose group (G4), treatment related histological findings, namely, focal to multifocal chronic inflammatory changes in lungs (males-7/10 & 5/10-females), focal, multifocal to single cell multifocal necrosis in liver (females-5/10, males-1/10), and multifocal macrophage aggregates, were observed in mesenteric lymph nodes (10/10-females) respectively. Since the treatment related changes of the above-mentioned tissues were observed in high dose group (G4), the evaluation of the lungs (males & females), liver and mesenteric lymph nodes (females) was extended to lower dose groups (G2 & G3) and recovery groups (G1R & G4R).

The lungs of both sexes of high dose recovery (males-2/5, females-3/5), mid dose (males-2/10, females-3/10), and low dose (females-1/10) group showed focal to multifocal chronic inflammatory lesions. However, treatment related changes in liver (multifocal single cell necrosis) were limited to mid dose (females-1/10) and high dose recovery group (females-3/5) and changes in mesenteric lymph nodes (multifocal aggregates of macrophages in cortex and paracortex) to low dose (females-2/10), mid dose (females-9/10), and high dose recovery (females-5/5) groups.

## 4. Discussion

Experimental results from the in vitro genotoxicity studies, bacterial reverse mutation test, chromosomal aberration and micronucleus test suggest the ubiquinol acetate to be nongenotoxic with no clastogenic or aneugenic effects at the tested dose levels. Similarly, nongenotoxic results were observed for ubiquinol in genotoxicity studies by Mitsuaki [14].

Dose confirmation and homogeneity analysis of ubiquinol acetate formulation prior to treatment (day 0) and at week 7 and week 13 of the study revealed the prepared formulations were homogenous at all the three layers (top, middle, and bottom) and concentrations of the active ingredient in the formulation were within the acceptable limits. Hence the prepared formulation procedure was adequate and confirmed that the accurate concentration of the test item in the formulation was administered to all the respective groups in the study.

Subchronic repeated dose toxicity study of ubiquinol acetate daily once for 90 days consecutively by oral gavage in Sprague Dawley rats showed no adverse clinical signs of toxicity and mortality/morbidity. There were no statistically significant and treatment related adverse effects on body weight, body weight gain, feed consumption, ophthalmological examination, neurological/functional examination, and urine parameters analyzed. Similar findings were reported by Fu [15] and Hatakeyama [16] in the oral subacute toxicity study.

Statistical significant changes in hemoglobin hematocrit, MCV, MCHC, and blood clotting time were observed. However, the significant changes were found to be non-dose-dependent, observed only in one sex, and no changes were observed in the correlated parameters (hemoglobin, RBC, and hematocrit) and there were not also any histological changes. Hence, these variations were considered to be incidental and not treatment related. All other parameters analyzed in male and females were comparable with the concurrent control group. The findings of general condition and appearance of the animals, clinical signs, body weight, feed consumption, neurological or functional observations, ophthalmoscopy and hematology in the present study were found in accordance with Hatakeyama and Kitano [14, 16].

Clinical chemistry parameters analyzed revealed significant increase (1.5-2 folds) in AST and ALT in high dose (600 mg/kg body weight) females of main study and recovery groups which further correlated with gross, histological changes. Hence the changes can be considered as treatment related adverse effects observed in only females. Increase in liver enzymes (ALT, AST) was also reported by Hatakeyama in female rats administered with ubiquinol at and above 300 mg/kg body weight for 13 weeks [16]. However, protective or beneficial effects of Coenzyme Q10 on liver were reported by Jiménez-Santos [17] and Esfahani et al. [18] at lower dose levels in rats. Significant increase in calculated globulin (G4M), bilirubin (G4M), total protein (G3M, G4M), and creatinine (G2M, G4RF) and statistical significant decrease in sodium (G4RM) and albumin (G4RF) were either non-dose-dependent, observed only in one sex, no correlation with histological changes or the changes not observed in the recovery groups. Hence, these variations were considered to be incidental and not treatment related.

Significant increase in absolute and relative weights of thymus, kidneys, prostate, seminal vesicles, and coagulating gland was considered as incidental due to lack of dose-dependency and lack of correlated histological changes. The gross pathological lesions observed in lungs as small yellow foci were correlating histologically with focal to multifocal areas of chronic inflammation. The cell infiltration was predominantly mononuclear with focal to multifocal in distribution and severity ranges from minimal to severe in lungs. The histopathological lesion observed was well correlated with small yellow foci at gross necropsy examination. The inflammatory lesions were also present in males and females of high dose recovery group at the end of 14 days of recovery period and also evaluation of lower dose groups showed similar lesion although the incidences were less.

Similar inflammatory lesion was reported earlier by Mitsuaki [14] and Hatakeyama [16] stated that chronic inflammation of the lungs could be due to aspiration of the test item into the lung during administration. Williams [19] reported similar unusual and dose related respiratory symptoms on repeated dosing of ubiquinone orally for 52-weeks in rats when corn oil was used as vehicle and concluded that the findings in the nasal turbinates and lungs were not systemically mediated toxic responses to test item. The findings of Willams were well supported by Siegrid [20] who also reported that the physiochemical properties of the test item may increase risk of respiratory effects due to gavage reflex after oral administration. The chronic inflammatory changes observed in lungs of treated animals in the present study were considered due to gavage reflex related and not direct toxicological insult by the test item.

Small white to yellow irregular foci in liver of female high dose groups were well correlated with focal necrotic area on histological evaluation. Treatment related adverse hepatocellular necrosis (single cell or multiple cells) in the high dose females was minimal to mild in severity and focal to multifocal in distribution. The liver lesion in females was well correlated with small white to yellow irregular areas with gross necropsy findings and also significant increase in AST and ALT levels. Similar findings were reported by Mitsuaki [14] as microgranuloma, mild focal necrosis in liver when treated with ubiquinol at 300 mg/kg and above, and these changes were thought to be the result of excess uptake of ubiquinol in the liver exceeding the capacity of adaptive response. Liver of recovery group females showed persistence of single cell necrosis with significant high level of ALT at the end of recovery period. However, one female from mid dose and one male from high group showed similar lesion with no significant change in liver enzymes and hence were considered to be incidental. The liver lesion at 600 mg/kg with significant increase in liver enzymes and its persistence at the end of recovery period is considered to be adverse.

Aggregates of macrophages in mesenteric lymph nodes were observed in all the females of main study and recovery high dose group with minimal to mild and multifocal in distribution in cortex and paracortex. The lesion was also observed in low and mid dose group in females with reduced severity as compared to the high dose group. Similar lesion was earlier reported as aggregates for foamy histiocytes by researchers. This finding was considered as adaptive response and nonadverse as it is unlikely to progress to an adverse condition with time to cause any functional impairment [21]. On histopathological examination, lungs, liver, and mesenteric lymph nodes were considered as the target organs. All other histopathological findings were sporadic in nature, comparable with concurrent control group, and hence considered incidental to the age of Sprague Dawley rats used in the study.

However, Honda et al. reported no changes with regard to body weight, food consumption, ophthalmoscopy, hematology, blood biochemistry, necropsy, organ weights, or histopathology at highest dose of 1200 mg/kg bw/day for 13 weeks oral CoQ10 administration in rats [22].

In summary, genotoxic studies of ubiquinol acetate by bacterial reverse mutation test in* Salmonella typhimurium *strains, in vitro micronucleus and in vitro chromosome aberration in CHO-K1 cell line, revealed the test item to be nongenotoxic with no clastogenic or aneugenic effects. The No-Observed-Adverse-Effect Level (NOAEL) of ubiquinol acetate when administered by oral gavage to Sprague Dawley rats for 90 consecutive days could be determined as 600 mg/kg body weight for males and 300 mg/kg body weight for females under the experimental conditions and doses employed. The NOAEL for ubiquinol acetate in female rats was lower than male rats which might be due to higher sensitivity to the accumulation of ubiquinol in the liver of female rats, a trend that might results from gender-dependent differences in the expression of hepatic enzymes. The liver changes might be due to excessive uptake of administered ubiquinol which have exceeded the adaptive capacity of the liver.

Human recommended dose of CoQ10 is 100-300 mg/day. Considering the highest human dose of 300 mg/day (5 mg/kg body weight) as a dietary supplement, a safety factor of 60-120 folds was found to be safer in humans. Based on the results of the present subchronic and genotoxicity studies, the chronic use of ubiquinol acetate as dietary supplements in humans is expected to be safe.

## Figures and Tables

**Table 1:** Stability of ubiquinol and ubiquinol acetate.

Compound	HPLC Purity (%)		
	Initiation	1^st^ month	3^rd^ month
Ubiquinol	98.00	53.00	22.00

Ubiquinol acetate	99.50	99.45	99.49

**Table 2:** Specifications & certificate of analysis of the ubiquinol acetate.

Sl.	Tests	Results	Limits
No.			
1	Appearance	Off-white solid	White to off-white solid

2	Melting range	46.7°C to 48.4°C	46°C to 52°C

3	Solubility	Very soluble in toluene and ethyl acetate	Very soluble in toluene and ethyl acetate
		Freely soluble in hexane	Freely soluble in hexane
		Sparingly soluble in ethanol	Sparingly soluble in ethanol
		Vey slightly soluble in methanol	Very slightly soluble in methanol
		Practically insoluble in water	Practically insoluble in water

4	Identification by IR	Complies	Should comply with that of standard

5	Purity by HPLC (area %)	98.2 %	Not less than 98.0 %
	Single largest unspecified impurity	RRT at 1.27=0.64%	Not more than 1.0 %
	Ubiquinone	0.19 %	Not more than 1.0 %

6	Assay (By HPLC) (% w/w)	99.1 %	Between 98.0% and 102.0%

7	Water Content by KF	0.30 %	Not more than 0.5 %

8	Residue on Ignition	0.02 %	Not more than 0.2 %

9	Heavy Metal	Less than 10 ppm	Not more than 10 ppm

10	Residual solvent by GC-HS		
	Ethanol	89 ppm	Not more than 500 ppm
	Pyridine	1 ppm	Not more than 200 ppm
	n-Heptane	8 ppm	Not more than 100 ppm

11	Acetic acid contents by GC	Not detected	Not more than 500 ppm

12	Microbial Analysis		
	TAMC	Nil	Not more than 100 CFU/g
	TYMC	Nil	Not more than 10 CFU/g
	*E. Coli*	Absent	Should be absent for 10 g
	Salmonella	Absent	
	Pseudomonas	Absent	
	Staphylococcus	Absent	

**Table 3:** Dose confirmation & homogeneity analysis.

Day of Dose Formulation used for administration	% Recovery of analysed concentration								
	(Dose Confirmation)								
	Low dose			Mid dose			High dose		
	(37.5 mg/mL)			(75.0 mg/mL)			(150.0 mg/mL)		

Day 0	104.15			104.28			101.31		
Day 49	96.74			97.58			98.10		
Day 90	94.94			95.62			95.68		

Day of Dose Formulation used for administration	% Recovery of analysed concentration								
	(Homogeneity)								
	Low dose			Mid dose			High dose		
	(37.5 mg/mL)			(75.0 mg/mL)			(150.0 mg/mL)		
	Top	Middle	Bottom	Top	Middle	Bottom	Top	Middle	Bottom

Day 0	102.32	103.30	103.39	103.40	104.42	104.84	106.28	107.59	105.63
Day 49	98.07	97.82	96.33	98.42	98.88	98.61	98.68	99.70	98.87
Day 90	96.06	96.11	93.87	96.19	96.86	97.11	97.57	96.78	96.56

Note: acceptable limits of % recovery: from 85 to 115 %.

**Table 4:** Cytogenetic analysis of cho-k1 cells treated with ubiquinol acetate in absence and presence of exogenous metabolic activation.

Treatment^a^ (*μ*g/mL)	Metabolic Activation	Treatment Time (hours)	Cytotoxicity^b^ (%)	Cells Scored	Normal Cells	Aberrant Cells	% Aberrant cells^c^	Total No. of Aberrations	Aberrations Per Cell
Vehicle^d^	Absence	4	0.00	300	297	3	1.00	3	0.010
MMC 0.4	Absence	4	14.97	50	21	29	58.00^e^	36	0.720
15.63	Absence	4	2.14	300	298	2	0.67	2	0.007
31.25	Absence	4	4.28	300	297	3	1.00	3	0.010
62.50	Absence	4	-3.21	300	298	2	0.67	2	0.007

Vehicle^d^	Presence	4	0.00	300	296	4	1.33	4	0.013
CP 5.0	Presence	4	13.37	300	275	25	8.33^e^	34	0.113
15.63	Presence	4	1.60	300	297	3	1.00	3	0.010
31.25	Presence	4	5.35	300	294	6	2.00	6	0.020
62.500	Presence	4	6.95	300	295	5	1.67	5	0.017

Vehicle^d^	Absence	24	0.00	300	298	2	0.67	2	0.007
MMC 0.4	Absence	24	13.37	50	14	36	72.00^e^	65	1.300
15.63	Absence	24	-6.95	300	296	4	1.33	4	0.013
31.25	Absence	24	-6.95	300	298	2	0.67	2	0.007
62.50	Absence	24	-2.14	300	297	3	1.00	3	0.010

^a^CHO-K1 cells treated with vehicle/control/test item at 37°C.

^b^Cytotoxicity (%) determined by comparing the relative increase in cell count of treated cultures to vehicle cultures.

^c^Cells with only gaps were considered as normal cells.

^d^1% acetone, MMC: mitomycin C and CP: cyclophosphamide.

^e^Statistically significant from vehicle control at p<0.05 by Fisher's exact test.

**Table 5:** In vitro micronucleus test for ubiquinol acetate in cho-k1 cells.

SL No.	Treatment	Total Micronuclei with Binucleated scored	Total Binucleated scored	% Micronuclei
*Treatment in absence of S9 (4 h)*				
1	Media control (HAM's F12-K)	12	2000	*0.60*
2	Vehicle control (1 % Acetone)	17	2000	*0.85*
3	Mitomycin C at 0.5 *μ*g/mL	188	2000	*9.40∗*
4	Ubiquinol acetate at 15.625 *μ*g/mL	14	2000	*0.70*
5	Ubiquinol acetate at 31.25 *μ*g/mL	14	2000	*0.70*
6	Ubiquinol acetate at 62.5 *μ*g/mL^*a*^	18	2000	*0.90*
*Treatment in absence of S9 (24 h)*				
7	Media control (HAM's F12-K)	16	2000	*0.80*
8	Vehicle control (1 % Acetone)	9	2000	*0.45*
9	Mitomycin C at 0.5 *μ*g/mL	213	2000	*10.65∗*
10	Ubiquinol acetate at 15.625 *μ*g/mL	17	2000	*0.85*
11	Ubiquinol acetate at 31.25 *μ*g/mL	12	2000	*0.60*
12	Ubiquinol acetate at 62.5 *μ*g/mL^*a*^	11	2000	*0.55*
*Treatment in presence of S9 (4 h)*				
13	Media control (HAM's F12-K)	12	2000	*0.60*
14	Vehicle control (1 % Acetone)	10	2000	*0.50*
15	Cyclophosphamide at 0.5 *μ*g/mL	144	2000	*7.20∗*
16	Ubiquinol acetate at 15.625 *μ*g/mL	17	2000	*0.85*
17	Ubiquinol acetate at 31.25 *μ*g/mL	13	2000	*0.65*
18	Ubiquinol acetate at 62.5 *μ*g/mL^*a*^	13	2000	*0.65*

‘*a*' least insoluble concentration; *∗* % micronuclei is statistically increased compared to the vehicle control at p <0.05.

**Table 6:** Effect of ubiquinol acetate on mean body weight gain (%).

Duration (Weeks)	Sex	Group & Dose (mg/kg body weight)					
		G1	G2	G3	G4	G1R	G4R
		0	150	300	600	0	600
1	Male	33.57 ± 2.78	32.07 ± 3.25	32.38 ± 3.03	33.89 ± 4.74	33.79 ± 2.81	34.56 ± 1.73
	Female	21.66 ± 5.11	18.55 ± 5.12	18.41 ± 4.42	20.34 ± 7.92	17.92 ± 4.32	21.23 ± 4.89

5	Male	117.76 ± 8.92	111.86 ± 20.18	114.20 ± 9.91	117.73 ± 19.00	117.59 ± 18.68	119.14 ± 5.95
	Female	53.21 ± 7.98	49.24 ± 6.68	52.34 ± 6.31	56.37 ± 8.10	55.15 ± 12.11	57.96 ± 9.53

9	Male	155.19 ± 13.63	144.19 ± 28.58	148.05 ± 12.17	155.12 ± 26.99	151.72 ± 25.47	150.03 ± 17.40
	Female	79.38 ± 12.25	73.12 ± 9.25	82.19 ± 11.95	77.60 ± 9.00	84.14 ± 14.98	83.03 ± 11.99

13	Male	174.36 ± 16.57	166.61 ± 33.70	168.17 ± 12.89	174.14 ± 31.09	168.81 ± 24.19	172.71 ± 21.19
	Female	85.17 ± 15.08	78.90 ± 12.56	86.19 ± 13.51	84.46 ± 11.19	88.71 ± 20.10	97.93 ± 15.37

15	Male	-	-	-	-	175.10 ± 23.79	179.42 ± 20.88
	Female	-	-	-	-	87.00 ± 20.58	97.85± 10.41

Mean ± SD for 10 rats/sex/group (G1, G2, G3, G4) and 5 rats/sex/group (G1R, G4R).

**Table 7:** Effect of ubiquinol acetate on hematological parameters in male rats.

Parameters	Units	Group & Dose (mg/kg b.w.)					
		G1,	G2,	G3,	G4,	G1R,	G4R,
		0	150	300	600	0	600
Total WBC count	10^3^/*μ*l	6.50 ± 0.99	6.79 ± 0.86	6.76± 1.03	6.63 ± 1.38	6.00±1.18	7.25±1.72

Total RBC count	10^6^/*μ*l	9.04 ± 0.51	9.11 ± 0.34	8.83± 0.41	8.64± 0.33	8.60±0.70	8.61±0.6

Hemoglobin	g/dL	15.62±0.48	15.58±0.38	15.64±0.46	14.85±0.61 ^*∗*^	15.90±0.93	15.42±0.76

Hematocrit	%	48.22±1.97	48.48±1.74	48.46±1.78	45.67±2.11 ^*∗*^	46.34±3.56	45.2±2.93

MCV	fL	53.39±1.92	53.28±2.94	54.94±1.52	52.92±3.01	53.90±1.91	52.54±1.83

MCH	pg	17.28±0.72	17.12±0.84	17.72±0.61	17.22±1.00	18.54±0.7	17.94±0.63

MCHC	g/dL	32.39±0.67	32.15±0.59	32.28±0.62	32.54±0.64	34.40±0.58	34.16±0.64

Neutrophils	10^3^/*μ*l	1.02±0.26	1.35±0.49	1.41±0.51	1.32±0.72	0.98±0.36	1.07±0.27

Lymphocytes	10^3^/*μ*l	5.07±0.88	5.01±0.75	4.95±0.56	4.91±1.04	4.64±0.92	5.78±1.40

Monocytes	10^3^/*μ*l	0.21±0.08	0.18±0.05	0.18±0.06	0.17±0.07	0.18±0.06	0.22±0.12

Eosinophils	10^3^/*μ*l	0.11±0.04	0.13±0.04	0.11±0.05	0.11±0.04	0.15±0.04	0.13±0.05

Basophils	10^3^/*μ*l	0.01±0.00	0.01±0.01	0.01±0.00	0.01±0.00	0.01±0.01	0.01±0.00

LUC	10^3^/*μ*l	0.09±0.03	0.11±0.04	0.09±0.05	0.11±0.05	0.03±0.03	0.05±0.03

Platelet count	10^3^/*μ*l	777.0±83.43	637.30±146.39	688.90±185.00	845.80±105.67	732.20±154.39	931.40±123.82

Blood clotting time	Seconds	154.20±11.64	165.10±23.39	165.80±23.72	154.90±27.74	169.00±28.52	164.60±27.41

Mean ± SD for 10 rats/sex/group (G1, G2, G3, G4) and 5 rats/sex/group (G1R, G4R);* * ^*∗*^: statistically significant compared to the control group (p < 0.05).

**Table 8:** Effect of ubiquinol acetate on hematological parameters in female rats.

Parameters	Units	Group & Dose (mg/kg b.w.)					
		G1,	G2,	G3,	G4,	G1R,	G4R,
		0	150	300	600	0	600
Total WBC count	10^3^/*μ*l	5.04±1.49	4.25 ± 0.99	5.13± 1.16	5.49±2.89	5.03±1.53	5.33±1.58

Total RBC count	10^6^/*μ*l	8.06±0.33	8.31 ± 0.30	8.09± 0.27	8.08± 0.35	8.18±0.32	7.94±0.24

Hemoglobin	g/dL	14.88±0.58	15.17±0.46	14.65±0.39	14.76±0.54	15.84±0.29	15.54±0.39

Hematocrit	%	45.24±1.86	45.10±1.04	43.55±0.92 ^*∗*^	43.54±1.80 ^*∗*^	45.42±1.86	43.48±1.00

MCV	fL	56.12±1.02	54.32±1.29 ^*∗*^	53.88±1.85 ^*∗*^	53.94±0.69 ^*∗*^	55.50±0.91	54.8±1.80

MCH	pg	18.47±0.31	18.27±0.42	18.14±0.71	18.31±0.46	19.36±0.47	19.6±0.24

MCHC	g/dL	32.90±0.31	33.66±0.44 ^*∗*^	33.62±0.52 ^*∗*^	33.95±0.65 ^*∗*^	34.90±0.86	35.76±0.85

Neutrophils	10^3^/*μ*l	0.99±0.51	0.72±0.17	1.03±0.49	1.35±1.27	0.62±0.26	0.95±0.42

Lymphocytes	10^3^/*μ*l	3.75±1.04	3.28±0.83	3.81±0.82	3.81±1.90	4.13±1.54	4.05±1.20

Monocytes	10^3^/*μ*l	0.12±0.03	0.11±0.04	0.12±0.03	0.14±0.06	0.11±0.05	0.16±0.04

Eosinophils	10^3^/*μ*l	0.11±0.06	0.07±0.02	0.10±0.07	0.11±0.08	0.13±0.09	0.12±0.06

Basophils	10^3^/*μ*l	0.01±0.01	0.00±0.00	0.01±0.01	0.01±0.01	0.01±0.01	0.01±0.00

LUC	10^3^/*μ*l	0.06±0.02	0.07±0.03	0.07±0.02	0.08±0.04	0.03±0.01	0.04±0.01

Platelet count	10^3^/*μ*l	783.10±190.59	790.40±263.71	691.10±185.00	834.80±295.27	934.6±240.07	858.4±291.48

Blood clotting time	Seconds	155.80±14.80	154.70±14.58	158.30±16.23	144.50±24.34	152.40±16.02	214±33.94 ^*∗*^

Mean ± SD for 10 rats/sex/group (G1, G2, G3, G4) and 5 rats/sex/group (G1R, G4R); ^*∗*^: statistically significant compared to the control group (p < 0.05).

**Table 9:** Effect of ubiquinol acetate on clinical chemistry parameters in male rats.

Parameters	Units	Group & Dose (mg/kg b.w.)					
		G1,	G2,	G3,	G4,	G1R,	G4R,
		0	150	300	600	0	600
Glucose	mg/dL	129.70±18.00	119.00±11.96	122.10±7.68	138.00±22.9	132.40±10.36	125.2±12.62

Blood Urea Nitrogen	mg/dL	16.30±1.77	15.80±2.04	15.50±2.17	17.50±2.42	11.80±0.45	13.40±2.51

Creatinine	mg/dL	0.31±0.05	0.34±0.08	0.38±0.05^*∗*^	0.37±0.05	0.28±0.05	0.30±0.04

Cholesterol	mg/dL	57.60±8.71	57.90±7.88	57.5±6.79	64.00±11.32	68.60±9.07	68.60±8.08

TGL	mg/dL	47.80±13.42	41.20±16.2	43.40±10.46	40.60±8.72	52.00±9.82	50.6±14.42

Total Protein	g/dL	6.64±0.25	6.74±0.13	6.96±0.22^*∗*^	7.17±0.25^*∗*^	6.84±0.13	6.78±0.08

Albumin	g/dL	1.22±0.08	1.25±0.1	1.31±0.12	1.23±0.11	1.22±0.08	1.18±0.13

Total Bilirubin	mg/dL	0.06±0.05	0.10±0.05	0.08±0.04	0.11±0.03^*∗*^	0.10±0.00	0.04±0.05

AST	U/L	68.20±17.63	79.80±22.76	67.70±11.80	81.8±20.32	67.60±2.19	70.00±10.84

Calcium	mg/dL	9.26±0.35	9.26±0.27	9.31±0.39	9.11±0.4	9.62±0.22	9.80±0.35

Phosphorus	mg/dL	5.72±0.48	5.64±0.43	5.70±0.29	5.78±0.15	5.90±0.12	6.12±0.45

ALP	U/L	98.40±19.1	93.50±15.88	94.20±13.25	108.9±21.34	81.80±10.64	77.60±5.37

ALT	U/L	20.00±4.06	24.10±8.41	20.10±3.48	25.70±7.10	22.60±2.51	26.40±6.11

GGT	U/L	2.90±1.37	2.10±0.74	3.00±1.33	2.70±0.82	6.20±0.84	4.80±1.92

Sodium	mmol/L	135.19±1.32	134.40±1.05	136.09±1.7	134.16±1.42	136.94±0.76	135.7±0.61^*∗*^

Potassium	mmol/L	4.19±0.24	4.23±0.35	4.14±0.25	4.32±0.16	4.29±0.17	4.12±0.38

Chloride	mmol/L	107.13±1.19	107.03±0.78	107.53±0.84	107.78±1.85	105.60±0.82	105.22±0.72

Globulin	g/dL	5.42±0.30	5.49±0.18	5.65±0.17	5.94±0.31^*∗*^	5.62±0.16	5.60±0.16

Mean ± SD for 10 rats/sex/group (G1, G2, G3, G4) and 5 rats/sex/group (G1R, G4R);* *^*∗*^: statistically significant compared to the control group (p < 0.05).

**Table 10:** Effect of ubiquinol acetate on clinical chemistry parameters in female rats.

Parameters	Units	Group & Dose (mg/kg b.w.)					
		G1,	G2,	G3,	G4,	G1R,	G4R,
		0	150	300	600	0	600
Glucose	mg/dL	124.50±25.44	105.90±17.91	116.3±12.03	111.40±12.61	122.2±8.11	116.20±9.78

Blood Urea Nitrogen	mg/dL	18.20±2.30	19.50±2.22	20.50±3.44	21.30±3.83	11.20±3.96	13.20±2.77

Creatinine	mg/dL	0.46±0.10	0.44±0.07	0.49±0.10	0.49±0.05	0.27±0.02	0.34±0.05^*∗*^

Cholesterol	mg/dL	86.70±18.78	91.60±17.99	87.40±22.80	90.20±19.87	103.40±9.86	120.20±23.83

TGL	mg/dL	31.90±11.94	34.90±5.59	31.20±7.94	34.10±8.18	31.80±9.20	25.60±4.93

Total Protein	g/dL	6.92±0.39	7.02±0.37	6.78±0.27	7.16±0.46	6.66±0.26	6.66±0.48

Albumin	g/dL	1.53±0.16	1.56±0.13	1.41±0.13	1.47±0.30	1.40±0.19	1.12±0.19^*∗*^

Total Bilirubin	mg/dL	0.11±0.06	0.08±0.09	0.07±0.05	0.11±0.06	0.10±0.07	0.06±0.05

AST	U/L	71.10±15.07	87.80±34.78	89.80±19.10	118.20±42.65^*∗*^	70.60±16.04	82.00±12.88

Calcium	mg/dL	9.80±0.39	9.90±0.22	9.39±0.28	9.28±1.66	9.02±0.31	9.20±0.33

Phosphorus	mg/dL	5.24±0.67	4.99±0.47	5.44±0.53	5.28±0.48	5.10±0.31	5.22±0.28

ALP	U/L	64.30±11.45	66.40±16.13	77.20±15.40	83.30±33.68	40.00±10.56	45.60±4.88

ALT	U/L	13.10±2.60	20.30±6.48	23.10±6.61^*∗*^	31.30±14.67^*∗*^	16.60±3.05	21.80±2.95^*∗*^

GGT	U/L	2.40±1.26	2.30±1.34	3.00±0.82	2.20±1.48	5.40±1.34	5.20±1.30

Sodium	mmol/L	135.14±1.78	135.84±1.55	135.18±1.56	136.30±2.02	137.52±0.76	136.92±0.36

Potassium	mmol/L	4.00±0.34	3.76±0.24	3.87±0.30	3.87±0.27	4.16±0.25	4.11±0.33

Chloride	mmol/L	107.81±1.41	107.77±1.38	107.23±1.27	108.72±1.62	107.60±0.74	107.58±0.86

Globulin	g/dL	5.35±0.32	5.46±0.33	5.35±0.22	5.69±0.35^*∗*^	5.26±0.18	5.54±0.47

Mean ± SD for 10 rats/sex/group (G1, G2, G3, G4) and 5 rats/sex/group (G1R, G4R);  ^*∗*^: statistically significant compared to the control group (p < 0.05).

**Table 11:** Effect of ubiquinol acetate on absolute organ weights.

Organ	Sex	Group & Dose (mg/kg b.w.)					
		G1,	G2,	G3,	G4,	G1R,	G4R,
		0	150	300	600	0	600
Kidneys	Male	3.5127±0.3930	3.3681±0.4378	3.4254±0.6575	3.6245±0.3467	2.9478±0.3413	3.4107±0.3825^*∗*^
	Female	2.0625±0.3258	1.8507±0.1611	1.9465±0.1566	1.9238±0.1899	1.9870±0.2523	2.1070±0.2536

Adrenals	Male	0.0641±0.0142	0.0721±0.0184	0.0650±0.0170	0.0665±0.0148	0.0451±0.0096	0.0575±0.0074
	Female	0.0716±0.0089	0.0743±0.0135	0.0749±0.0090	0.1521±0.2338	0.0712±0.0140	0.0732±0.0095

Spleen	Male	0.9394±0.1453	0.8933±0.1381	0.9379±0.1544	1.0691±0.2414	0.7964±0.1151	0.9713±0.1424
	Female	0.6409±0.0939	0.5568±0.0705	0.6637±0.0759	0.7218±0.1514	0.6675±0.1877	0.7605±0.1765

Heart	Male	1.6051±0.1723	1.6878±0.2243	1.6721±0.1998	1.6202±0.1868	1.5127±0.1457	1.5358±0.0668
	Female	1.0702±0.1039	0.9925±0.0573	0.9922±0.0791	1.0658±0.0987	1.0543±0.1028	1.0918±0.0728

Liver	Male	17.5704±2.2358	16.2747±2.6032	16.6456±3.3436	18.0420±2.9797	14.4688±1.7072	15.9630±1.6045
	Female	9.1951±1.1801	8.3073±1.0307	8.3006±0.6693	9.2363±1.1137	8.9485±1.1943	9.3907±0.7840

Thymus	Male	0.4835±0.1050	0.5787±0.0902	0.5553±0.1422	0.6622±0.1336^*∗*^	0.4977±0.0706	0.4989±0.1741
	Female	0.4626±0.1532	0.3649±0.0731	0.4044±0.0869	0.3860±0.0728	0.4369±0.1188	0.4679±0.1589

Brain	Male	2.3552±0.1246	2.3494±0.1032	2.2999±0.1290	2.2327±0.1292	2.2089±0.1059	2.3116±0.0853
	Female	2.1233±0.1200	2.0894±0.1806	2.1484±0.0966	2.0909±0.1055	2.1446±0.1097	2.1692±0.0615

Testes	Male	4.0722±0.3766	4.1204±0.3059	3.9762±0.5078	4.0524±0.2852	3.7692±0.3181	3.9867±0.0434

Epididymides	Male	1.6995±0.2227	1.6857±0.1587	1.4988±0.2433	1.6142±0.1712	1.4772±0.961	1.5197±0.1067

PSVC	Male	3.6996±0.7613	3.3859±0.6118	3.3255±0.5844	3.1484±0.4997	2.6255±0.2869	3.4107±0.3825^*∗*^

Ovaries	Female	0.1454±0.0341	0.1217±0.0274	0.1289±0.0185	0.1337±0.0363	0.1291±0.0190	0.1348±0.0083

Uterus	Female	0.9388±0.3061	1.1497±0.5136	1.1038±0.5532	1.2858±0.7225	1.5083±0.9090	1.3469±0.6788

Mean ± SD for 10 rats/sex/group (G1, G2, G3, G4) and 5 rats/sex/group (G1R, G4R);  ^*∗*^: statistically significant compared to the control group (p < 0.05).

**Table 12:** Effect of ubiquinol acetate on relative organ weights (%).

Organ	Sex	Group & Dose (mg/kg b.w.)					
		G1,	G2,	G3,	G4,	G1R,	G4R,
		0	150	300	600	0	600
Kidneys	Male	0.7232±0.0792	0.7257±0.0882	0.7168±0.0889	0.7704±0.0929	0.6495±0.0499	0.7258±0.0518^*∗*^
	Female	0.7810±0.0837	0.7552±0.0768	0.7651±0.0862	0.7580±0.1307	0.7703±0.0721	0.7748±0.0747

Adrenals	Male	0.0131±0.0025	0.0155±0.0038	0.0138±0.0040	0.0140±0024	0.0100±0.0025	0.0124±0.0021
	Female	0.0276±0.0055	0.0303±0.0055	0.0294±0.0035	0.0618±0.0991	0.0266±0.0064	0.0271±0.0046

Spleen	Male	0.1931±0.0272	0.1931±0.0330	0.1975±0.0286	0.2250±0.0415	0.1757±0.0223	0.2093±0.0322
	Female	0.2434±0.0290	0.2269±0.0292	0.2603±0.0313	0.2798±0.0451	0.2194±0.0388	0.2791±0.0561

Heart	Male	0.3303±0.0308	0.3632±0.0429	0.3527±0.0385	0.3425±0.0270	0.3335±0.0201	0.3310±0.0237
	Female	0.4084±0.0452	0.4052±0.0318	0.3897±0.0395	0.4174±0.0539	0.3950±0.0448	0.4019±0.0164

Liver	Male	3.6062±0.3184	3.5019±0.5405	3.4752±0.4577	3.8245±0.6361	3.1830±0.1809	3.4402±0.3901
	Female	3.4989±0.3900	3.3898±0.4581	3.2648±0.3799	3.6217±0.5849	3.2776±03004	3.4542±0.1666

Thymus	Male	0.1000±0.0242	0.1244±0.0169	0.1186±0.0382	0.1403±0.0282^*∗*^	0.1098±0.0133	0.1075±0.0373
	Female	0.1736±0.0472	0.1478±0.0239	0.1574±0.0269	0.1508±0.0271	0.1587±0.0323	0.1730±0.0623

Brain	Male	0.4855±0.0356	0.5089±0.0549	0.4877±0.0564	0.4745±0.0420	0.4889±0.0389	0.4982±0.0333
	Female	0.8135±0.0990	0.8527±0.0879	0.8434±0.0579	0.8216±0.1089	0.8317±0.1305	0.8003±0.0493

Testes	Male	0.8381±0.0739	0.8934±0.1197	0.8371±0.0862	0.8593±0.0587	0.8314±0.0444	0.8588±0.0350

Epididymides	Male	0.3511±0.0544	0.3645±0.0447	0.3150±0.0395	0.3430±0.0436	0.3256±0.0340	0.3278±0.0328

PSVC	Male	0.7677±0.1872	0.7281±0.1176	0.6993±0.1007	0.6726±0.1355	0.5811±0.0714	0.7338±0.0763^*∗*^

Ovaries	Female	0.0554±0.0124	0.0497±0.0114	0.0504±0.0065	0.0519±0.0129	0.0483±0.0123	0.0498±0.0052

Uterus	Female	0.3569±0.1083	0.4671±0.2051	0.4354±0.2283	0.5023±0.2869	0.6827±0.5235	0.4912±0.2288

Mean ± SD for 10 rats/sex/group (G1, G2, G3, G4) and 5 rats/sex/group (G1R, G4R); ^*∗*^: statistically significant compared to the control group (p < 0.05).

**Table 13:** Summary of gross necropsy findings, males and females.

Organs	Group and Dose (mg/kg b.w.)											
Diagnoses	G1		G1R		G2		G3		G4		G4R	
	0		0		150		300		600		600	
	TS		TS		TS		TS		TS		TS	
	M	F	M	F	M	F	M	F	M	F	M	F
	[10]	[10]	[5]	[5]	[10]	[10]	[10]	[10]	[10]	[10]	[5]	[5]
*Liver *												
No Abnormality Detected	10	10	5	5	10	10	10	10	10	8	5	5
Small white to yellow irregular area, focal	0	0	0	0	0	0	0	0	0	2	0	0

*Lungs *												
No Abnormality Detected	10	10	5	5	10	10	10	10	8	7	5	4
Small yellow foci	0	0	0	0	0	0	0	0	2	3	0	1

Note: the numerical indicated under observation represents the number of animals with and without lesions; M: male; F: female; TS: terminal sacrifice.

**Table 14:** Summary of histopathological examination, males and females.

Organs	Group and Dose											
Diagnoses	G1		G2^*∗*^		G3^*∗*^		G4^*∗*^		G21R^*∗*^		G24R^*∗*^	
	0		150		300		600		0		600	
	TS		TS		TS		TS		TS		TS	
	M	F	M	F	M	F	M	F	M	F	M	F
	[10]	[10]	[10]	[10]	[10]	[10]	[10]	[10]	[5]	[5]	[5]	[5]
*Lungs*	(10)	(10)	(10)	(10)	(10)	(10)	(10)	(10)	(5)	(5)	(5)	(5)
Within normal limits	10	10	10	9	7	7	2	4	5	5	3	2
Inflammation, chronic focal/multifocal	0	0	0	1	2	3	7	5	0	0	2	3
Alveolar macrophages, multifocal	0	0	0	0	1	0	1	2	0	0	0	0

*Liver*	(10)	(10)	-	(10)	-	(10)	(10)	(10)	-	(5)	-	(5)
Within normal limits	7	8		10		9	8	5		4		2
Fatty change, multifocal	3	1		0		0	1	0		1		0
Necrosis, focal/multifocal	0	1		0		0	1	3		0		0
Necrosis, single cell, multifocal	0	0		0		1	0	2		0		3

*Lymph node, mesenteric*	(10)	(10)	-	(10)	-	(10)	(10)	(10)	-	(5)	-	(5)
Within normal limits	10	10		8		1	10	0		5		0
Aggregates, macrophages, multifocal	0	0		2		9	0	10		0		5

Note: the numerical indicated under observation represents the number of animals with and without lesions; M: male; F: female; TS: terminal sacrifice, *∗*: target organs evaluated based on the lesions observed in high dose main study group.

## Data Availability

Data Available on Request to the corresponding author.

## Abbreviations

ALP: Alkaline phosphatase
ALT: Alanine aminotransferase
AST: Aspartate aminotransferase
ATP: Adenosine triphosphate
GLP: Good laboratory practices
GGT: Gamma-glutamyl transferase
BUN: Blood urea nitrogen
NOAEL: No-Observed-Adverse-Effect Level
OECD: Organization for economic cooperation and development.

## References

[1] Kubo H., Fujii K., Kawabe T., Matsumoto S., Kishida H., Hosoe K. (2008). Food content of ubiquinol-10 and ubiquinone-10 in the Japanese diet. *Journal of Food Composition and Analysis*.

[2] Mattila P., Kumpulainen J. (2001). Coenzymes Q9 and Q10: Contents in foods and dietary intake. *Journal of Food Composition and Analysis*.

[3] Ernster L., Dallner G. (1995). Biochemical, physiological and medical aspects of ubiquinone function. *BBA - Molecular Basis of Disease*.

[4] Hosoe K., Kitano M., Kishida H., Kubo H., Fujii K., Kitahara M. (2007). Study on safety and bioavailability of ubiquinol (Kaneka QH™) after single and 4-week multiple oral administration to healthy volunteers. *Regulatory Toxicology and Pharmacology*.

[5] Varela-López A., Giampieri F., Battino M., Quiles J. L. (2016). Coenzyme Q and its role in the dietary therapy against aging. *Molecules*.

[6] Littarru G. P., Tiano L. (2007). Bioenergetic and antioxidant properties of coenzyme Q10: recent developments. *Molecular Biotechnology*.

[7] Greenberg S., Frishman W. H. (1990). Co-enzyme Q10: a new drug for cardiovascular disease. *Clinical Pharmacology and Therapeutics*.

[8] García-Corzo L., Luna-Sánchez M., Doerrier C. (2014). Ubiquinol-10 ameliorates mitochondrial encephalopathy associated with CoQ deficiency. *Biochimica et Biophysica Acta (BBA) - Molecular Basis of Disease*.

[9] Garrido-Maraver J., Cordero M. D., Oropesa-Ávila M. (2014). Coenzyme q10 therapy. *Molecular Syndromology*.

[10] Zhou S., Wolinsky I., Driskell J. A. (2004). Coenzyme Q10. *Nutritional Ergogenic Aids*.

[11] Boreková M., Hojerová J., Koprda V., Bauerová K. (2008). Nourishing and health benefits of coenzyme Q10 - a review. *Czech Journal of Food Sciences*.

[12] Matsuo K., Kasai K., Hosoe K., Funahashi I. (2016). Stability of ubiquinol-10 (reduced form of coenzyme Q10) in human blood. *Biomedical Chromatography*.

[13] Kothari S. C., Shivarudraiah P., Venkataramaiah S. B. (2011). Safety assessment of Cissus quadrangularis extract (CQR-300): subchronic toxicity and mutagenicity studies. *Food and Chemical Toxicology*.

[14] Kitano M., Watanabe D., Oda S. (2008). Subchronic oral toxicity of ubiquinol in rats and dogs. *International Journal of Toxicology*.

[15] Fu X., Ji R., Dam J. (2009). Acute, subacute toxicity and genotoxic effect of Bio-Quinone® Q10 in mice and rats. *Regulatory Toxicology and Pharmacology*.

[16] Hatakeyama S., Kawase S., Yoshimura I. (2006). Comparative oral toxicity of coenzyme Q10 and its (2Z)-isomer in rats: single and four-week repeated dose toxicity studies. *Journal of Nutritional Science and Vitaminology*.

[17] Jiménez-Santos M. A., Juárez-Rojop I. E., Tovilla-Zárate C. A. (2014). Coenzyme Q10 supplementation improves metabolic parameters, liver function and mitochondrial respiration in rats with high doses of atorvastatin and a cholesterol-rich diet. *Lipids in Health and Disease*.

[18] Esfahani S. A., Esmaeilzadeh E., Bagheri F., Emami Y., Farjam M. (2013). The effect of co-enzyme Q10 on acute liver damage in rats, a biochemical and pathological study. *Hepatitis Monthly*.

[19] Williams K. D., Maneke J. D., Abdelhameed M. (1999). 52-Week oral gavage chronic toxicity study with ubiquinone in rats with a 4-week recovery. *Journal of Agricultural and Food Chemistry*.

[20] Damsch S., Eichenbaum G., Tonelli A. (2011). Gavage-related reflux in rats: identification, pathogenesis, and toxicological implications (Review). *Toxicologic Pathology*.

[21] Thiel A., Braun W., Cary M. G. (2013). Calcium lignosulphonate: re-evaluation of relevant endpoints to re-confirm validity and NOAEL of a 90-day feeding study in rats. *Regulatory Toxicology and Pharmacology*.

[22] Honda K., Tominaga S., Oshikata T. (2007). Thirteen-week repeated dose oral toxicity study of coenzyme Q10 in rats. *Journal of Toxicological Sciences*.

